# How does mode of delivery associate with double burden of malnutrition among mother–child dyads?: a trend analysis using Bangladesh demographic health surveys

**DOI:** 10.1186/s12889-022-13660-5

**Published:** 2022-06-23

**Authors:** Tasmiah Sad Sutopa, Wasimul Bari

**Affiliations:** Department of Statistics, University of Dhaka, Dhaka, Bangladesh

**Keywords:** Double burden of malnutrition, Mode of delivery, Caesarean, Bangladesh, Trend

## Abstract

**Background:**

The simultaneity of undernourishment among child and overweight/obesity among mothers in lower-and-middle-income-countries (LMICs) introduces a new nutrition dilemma, known as double burden of malnutrition (DBM). Amidst of such paradox, the hike of caesarean section (CS) delivery is also triggering child undernutrition and maternal obesity. A gap of knowledge regarding the effect of mode of delivery on DBM still persists. The study aims to explore the association between DBM at household level and mode of delivery over time in LMICs.

**Method:**

The study used data from recent four consecutive waves of Bangladesh Demographic and Health Survey (BDHS) ranging from BDHS 2007 to BDHS 2017. It considered the mother–child pairs from data where mothers were non-pregnant women aged 15–49 years having children born in last 3 years preceding the survey. Bivariate analysis and Logistic Regression were performed to explore the unadjusted and adjusted effect of covariates on DBM. An interaction term of mode of delivery and survey year was considered in regression model.

**Results:**

The study evinces a sharp increase of DBM rate in Bangladesh from 2007 to 2017 (2.4% vs. 6.4%). The prevalence of DBM in household level among the children delivered by CS is more than two times of those born by normal delivery (8.2% vs. 3.5%). The multivariate analysis also indicates that the children born by CS delivery are more likely to be affected by DBM at household level significantly than those born by normal delivery in each waves. Moreover, the odds ratio (OR) of DBM at household is increased by 43% for one unit change in time for normal delivery whereas CS delivery births have 12% higher odds of DBM at household level with one unit change in time.

**Conclusion:**

The study discloses a drastic increase of rate of DBM among mother–child pairs over the time. It stipulates inflated risk of DBM at household with time for both mode of delivery but the children with CS delivery are at more risk to the vulnerability of DBM at household level. The study recommends a provision of special care to the mothers with CS delivery to reduce DBM at household.

## Introduction

Lower-and-middle-income-countries (LMICs) have been going through a nutrition transition due to rapid economic growth and technological advancement [1]. Though LMICs have a long history of acute malnutrition among children because of food insecurity, disease burden and other social and demographical constraints [1], the growing need of urban migration, lack of physical activity, escalation of sedentary life and broadening food supply are pushing the expansion of overweight and obesity among adult in these countries [1, 2]. Hence, LMICs are now struggling to manage the paradoxical situation arising from the simultaneity of undernourishment among children and overweight among adults [3]. The concurrent persistence of overweight among mothers and malnourishment among their children introduces a new nutrition reality named double burden of malnutrition (DBM), which imposes a challenging situation for the LMICs [4, 5]. While the world is promisingly heading towards achieving Sustainable Development goals (SDGs), especially eradicating all forms of malnutrition (Goal 2) and achieving assurance for healthy lives and well-being in all age groups (Goal 3) [6], it is highly needed to focus on DBM instead of addressing only one form of malnutrition such as undernourishment or obesity.

Globally, more than one-third of LMICs are going through a paradoxical situation with two extreme forms of malnutrition- undernutrition and overweight. Estimates from World Health Organization (WHO) depict that almost 2.3 billion children and adults are the victims of overweight whereas more than 150 million children are reported as stunted on a global premise [7]. Undernourishment among children is an apparent driving factor behind the increase of communicable diseases such as acute respiratory disease, malaria, diarrhea, etc. whereas uncontrolled obesity among the adult population is a leading promoter of non-communicable diseases (NCDs) like cardiovascular disease, high blood pressure, diabetes etc. [8]. Hence the puzzle of DBM can lead to the adverse effect of the double burden of disease with the simultaneous presence of NCDs and infectious diseases among the population [8]. Moreover, DBM also provokes an increase in health-care cost, depletion in productivity and deceleration in economic growth which perpetuates an intergenerational cycle of poverty and deteriorated health system [4].

Amid the upsurge of DBM as a new nutritional threat, experts are also concerned about the stark increase in caesarean section (CS) delivery, which causes many long and short-term adverse consequences on maternal as well as infant health [9]. In 1985, the international health community has drawn an ideal boundary for CS delivery rates ranging from 10 to 15%. Currently, one-fifth of births are delivered by CS which is beyond the safe limit [10]. The delivery through CS is also associated with different forms of undernourishment such as drastic weight loss and stunting among children which ultimately calls for impaired mental growth and lack of energy [9, 11, 12]. Moreover, mothers need to refrain from physical exercise during their postpartum period for a certain time after CS to avoid internal infections which may lead them to be overweight [13] and an initiation of vicious cycle of DBM may occur as a consequence.

Several studies have been conducted to understand the level and pattern of DBM in different LMICs around the world. A study conducted in South and Southeast Asian countries suggested that older maternal age and lower educational status are driving factors behind the increase in DBM [14]. Several studies argued that there exist strong evidences on the association between DBM and social-economic status [15, 16]. Popkin et al. stated in a study that the concurrence of rapid growth in adult obesity rate along with a slower pace in the reduction rate of undernourished children is exacerbating the problem of DBM at the household level in LMICs [17]. To the best of our knowledge, no studies till now have been conducted to examine the association between the DBM at the household level and CS delivery in LMICs.

This study mainly aims to explore the association between DBM at the household level and CS delivery in Bangladesh, an LMIC since 2015 [18]. The study will attempt to provide evidence on the effect of CS delivery on DBM among mother–child pairs at the household level in the context of Bangladesh by following the trend of DBM over a decade from 2007 to 2017 so that policymakers can plan proper interventions to face the current dilemmatic reality of nutrition transition in the country.

## Methodology

### Data

For the purpose of analysis, data were extracted from the last four Bangladesh Demographic Health Survey (BDHS) conducted in 2007, 2011, 2014 and 2017 and then combined. BDHS survey is a nationally representative survey that collects current information on the major indicators of maternal and child health-related issues. The survey was implemented by the National Institute of Population Research and Training (NIPORT), Health Education and Family Welfare Division of the Ministry of Health and Family Welfare. United States Agency for International Development (USAID) provided financial assistance in conducting the survey [19–22].

BDHS follows two stage stratified sampling plan where the enumeration areas from the Population and Housing Census of the People’s Republic of Bangladesh, provided by the Bangladesh Bureau of Statistics are considered as the primary sampling unit and a systemic sample of households within the survey is counted as the secondary sampling unit. The ever-married women in reproductive age are interviewed from the selected households in the sample for necessary information regarding maternal and child health indicators. The anthropometry measures of the respondents and their children under the age of five years are collected in these surveys [19–22]. The interviewers used lightweight SECA scale with a digital screen manufactured under the authority of UNICEF for measuring the weight. The height was measured by height boards specially produced by Shorr Production according to study settings. Recumbent length for children less than 2 years and standing height for the elder children are recorded in the survey. The detail of the survey methodology can be found elsewhere [19–22].

**Fig. 1:**
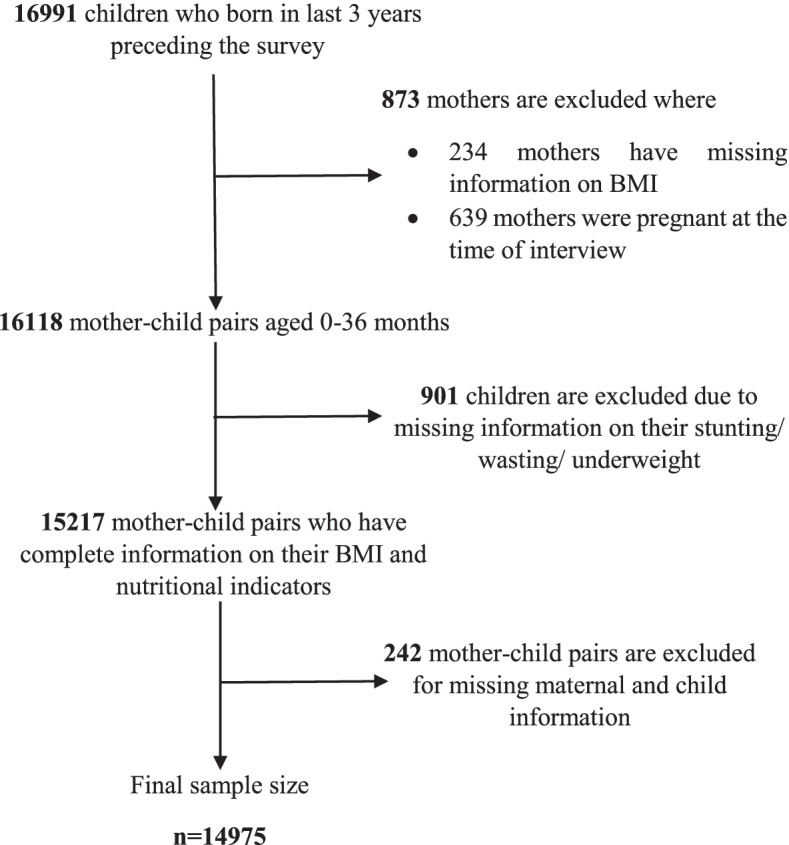
Flow chart for sample size selection

The study considered the mother–child pairs from four waves of BDHS survey where mothers are non-pregnant women aged 15–49 years and they had children who were born in the last 3 years preceding the survey. The details of the number of cases considered in this study along with the criteria that result in the exclusion of cases are explained in Fig. 1. After considering all desired criteria, we included 14,975 mother–child pairs combining the aforementioned BDHS surveys.

### Outcome variable

**Fig. 2:**
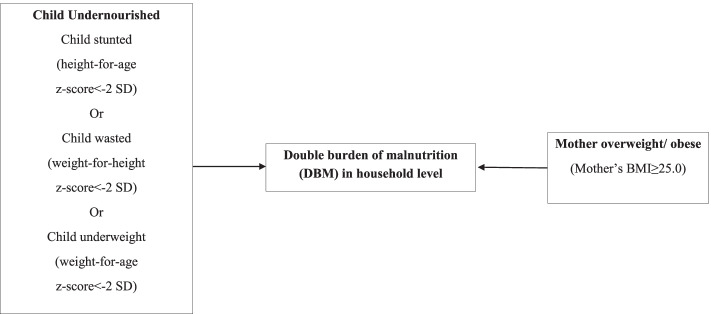
Diagram for definition of DBM

The binary outcome variable of interest in this study is the double burden of malnutrition (DBM) status at the household level defined considering the nutrition status of the mother and her child. The presence of DBM at the household level (taking value 1) arises if a mother is overweight or obese and her child is malnourished [5]. A mother is identified as overweight or obese if her BMI is 25 kg/m^2^ or more [23]. A child is considered to suffer from under-nutrition if s/he is stunted or wasted or underweight [24]. Stunting, wasting and underweight are assessed following the measurement of the WHO Child Growth (WHO) Standards reference population [25]. The definition of DBM is illustrated in Fig. 2.

### Independent variables

Several covariates are included in the study based on the suggestions of the previous studies. The prime focus of this study is on the mode of delivery which is categorized as “C-section” and “normal” based on the procedure followed during the child’s birth. The other independent variables that are included in the study are current breastfeeding status (yes, no), division (Barisal, Chattogram, Dhaka, Khulna, Rajshahi, Sylhet), place of residence (rural, urban), wealth index (poor, middle, rich), media exposure (yes, no), mother’s education level (no education, primary, secondary, higher), father’s education level (no education, primary, secondary, higher), mother’s working status (yes, no), mother’s decision-making capacity (yes, no), attitude towards violence (yes, no), mother’s age (15 to 24, greater than 24), received antenatal care (ANC) (yes, no), wanted child (yes, no), initiation of breastfeeding (within 1 h, after 1 h), child’s sex (female, male), birth order (first birth, otherwise), child’s age (in months) (< 18, 18 +), vitamin A consumption (yes, no).

It is to be noted that some variables are reconstructed to ensure consistency among variables’ categories in four consecutive surveys. We considered the categories of division as mentioned in BDHS 2007 whereas BDHS 2011 and BDHS 2014 consist of 7 categories for division and BDHS 2017–2018 subclasses division into 8 categories. Therefore, we merged Rangpur with Rajshahi for BDHS 2007, BDHS 2011, BDHS 2014 and BDHS-2017–18 and Mymensingh with Dhaka for BDHS 2017–18. To create the variable named “attitude towards violence” we considered respondent’s attitude towards wife beating being justified if she (1) burns food (2) argues with her husband (3) goes out without telling him, (4) neglects the children, and (5) refuses to have sex with him for BDHS 2011, BDHS 2014 and BDHS 2017–18. BDHS 2007 recorded the responses only for the first four reasons. If the respondent justifies at least one reason, she is considered in “yes” category, otherwise “no”. A woman is categorized as “yes” in the context of women’s decision-making capacity if she participates any of the following decisions on (1) her own health care, (2) major household purchases, and (3) visits to her family or relatives. If a mother received antenatal care at least once during her pregnancy is coded as “yes” for the variable “received antenatal care (ANC)”, otherwise the individual is coded as “no”. For constructing the variable “media exposure”, the respondent is categorized as “yes” if she is in touch with any of the following media at least once in every week (1) newspaper/ magazine, (2) radio, (3) television.

### Statistical methods

The study used the pooled dataset from the aforesaid BDHS surveys for examining the trend of DBM and the effect of mode of delivery on DBM by survey years. Firstly, the profile of background characteristics of the mother–child pairs is reported by survey years. Percentage distribution of DBM by several characteristics is depicted by survey years and latterly in the pooled dataset. Chi-square test is used in the pooled dataset to measure the association between DBM and the included independent variables. The independent variables that are significantly associated with DBM are considered further for regression analysis. Bivariate logistic regression analysis is used to pooled dataset to determine the adjusted association of covariates on DBM. The interaction effect between mode of delivery and survey year is allowed in regression model to assess the change in DBM at the household level by mode of delivery over time. The analysis is performed using SPSS version 22 and STATA statistical package version 12.

## Results

### Univariate analysis

The percentage distribution of selected characteristics of mother–child dyads is presented in Table 1 disintegrating the pooled data by survey years. The study included 2899, 3870, 3829 and 4377 mother–child pairs from BDHS 2007, BDHS 2011, BDHS 2014 and BDHS 2017 respectively.

**Table 1:** Percentage distribution of selected variables in the study over different survey years

		Survey Year				Pooled (*n* = 14,975)
		2007 (*n* = 2899)	2011 (*n* = 3870)	2014 (*n* = 3829)	2017 (*n* = 4377)	
DBM	Yes	2.4	3.4	5.1	6.4	4.5
	otherwise	97.6	96.6	94.9	93.6	95.5
Mode of Delivery	C-section	10.9	18.2	24.6	33.9	23.0
	Normal	89.1	81.8	75.4	66.1	77.0
Current Breastfeeding status	Yes	88.7	88.7	87.7	87.1	88.0
	No	11.3	11.25	12.3	12.9	12.0
Division	Barisal	12.8	10.8	12.2	10.9	11.6
	Chattogram	20.2	20.0	18.8	16.3	18.7
	Dhaka	22.0	16.2	18.0	25.9	20.6
	Khulna	12.4	12.9	11.9	10.7	11.9
	Rajshahi	17.6	25.7	25.4	22.6	23.1
	Sylhet	15.0	14.5	13.7	13.6	14.1
Place of Residence	Rural	63.2	67.7	68.0	66.3	66.5
	Urban	36.8	32.3	32.0	33.7	33.5
Wealth Index	Poor	38.6	39.6	39.3	42.1	40.1
	Middle	18.5	19.4	19.6	18.3	18.9
	Rich	42.9	41.0	41.2	39.7	41.0
Media Exposure	Yes	64.0	66.4	63.3	64.4	64.6
	No	36.0	33.6	36.7	35.6	35.4
Mother’s Educational Level	No education	23.1	15.9	13.1	6.3	13.8
	Primary	30.7	29.1	26.9	27.4	28.4
	Secondary	36.8	46.2	48.5	48.3	45.6
	Higher	9.3	8.8	11.5	18.0	12.3
Father’s Educational Level	No education	48.8	41.9	38.3	32.8	39.7
	Primary	31.0	37.2	38.7	42.6	38.0
	Secondary	6.4	6.6	7.6	5.6	6.5
	Higher	13.8	14.4	15.5	19.0	15.9
Mother’s Working Status	Yes	21.4	7.9	21.5	37.5	22.6
	No	78.6	92.1	78.5	62.5	77.4
Mother’s Decision-making Capacity	Yes	78.6	73.1	73.1	84.9	77.6
	No	21.4	26.9	26.9	15.1	22.4
Attitude towards Violence	Yes	32.3	32.2	27.9	17.8	26.9
	No	67.7	67.8	72.1	82.2	73.1
Mother’s Age	15 to 24	54.7	55.9	54.7	52.7	54.5
	Greater than 24	45.3	44.1	45.3	47.3	45.5
Received ANC	Yes	65.2	70.5	79.4	92.1	78.1
	No	34.8	29.5	20.6	7.9	21.9
Wanted Child	Yes	69.1	70.8	74.5	78.3	73.6
	No	30.9	29.2	25.5	21.7	26.4
Initiation of Breastfeeding	Within 1 Hour	56.3	52.4	48.8	39.3	51.6
	After 1 Hour	43.7	47.6	51.2	60.7	48.4
Child’s Sex	Female	49.9	49.4	48.1	47.3	48.5
	Male	50.1	50.6	51.9	52.7	51.5
Birth Order	First Birth	32.7	36.5	40.1	37.2	36.9
	Otherwise	67.3	63.5	59.9	62.8	63.1
Child’s Age	< 18	55.5	57.9	55.9	58.4	57.1
	18 +	44.5	42.1	44.1	41.6	42.9
Vitamin A Consumption	Yes	64.7	49.4	55.7	68.9	59.7
	No	35.3	50.6	44.3	31.1	40.3

It is vivid from Table 1 that there is a sharp increase in the prevalence of DBM in the household level over time. The rate of DBM in mother–child pairs almost tripled from 2007 to 2017 (2.4% vs. 6.4%). A hike of 42% relative increase in DBM is apparent from BDHS 2007 to BDHS 2011. The relative increase is paced to 50% from BDHS 2011 to BDHS 2014 and latterly lowers down the speed of relative increase to 25% from BDHS 2014 to BDHS 2017. Accordingly, the average relative increase is 38% which can bring about a hazardous boom in the prevalence of DBM in near future. Concurrently, the rate of CS delivery also reveals a steep rise over the years. Currently, one-third of the births in Bangladesh are delivered by CS Sect. (33.9%). From the first to last wave of the surveys considered in the study, the percentage of CS delivery increases 3 times (10.9% vs 33.9%). The average rate of increase among survey years is 45% that may lead to dramatic expansion of unnecessary CS delivery.

It is also found in Table 1 that 12% of the children considered in the study were not breastfed during the respective interview time. One-third of the cases considered in this study belong to urban areas of the country. Though the proportion of poor in the sample seems to be increasing over time (38.6% vs. 42.1%), the percentage of the sample exposed to media stalled around 64% over time. While most of the mothers in the pooled sample are educated up to secondary level (45.6%), two-fifth of fathers have no education. There is major progress in pthe ercentage of working mothers and enhancement of mother’s decision-making capacity from 2007 (21.4% and 78.6% respectively) to 2017 (37.5% and 84.9% respectively). About 27% of mothers justified their attitude toward violence. 55% of respondents in the pooled sample are young mothers. There is a dramatic increase in the percentage of ANC recipients from 2007 to 2017 (65.2% to 92.1%). The increase in the rate of wanted child (69.1% to 78.3%) indicates the fact that the couples are becoming more capable of utilizing family planning methods. The sex ratio in the pooled sample is 106 males per 100 females and 57% of the children in sample are aged below 18 months.

### Bivariate analysis

Table 2 is illustrating the percentage distribution of DBM by several background characteristics over time and in the combined sample also. The unadjusted association between the DBM and covariates is measured in pooled data by chi-square test.

**Table 2:** Prevalence of DBM by demographic, socio-economic and health related variables over the survey years

Variables and their Categories		Year				Overall	*p*-value
		**2007**	**2011**	**2014**	**2017**		
Mode of Delivery	C-section	7.0	7.7	8.3	8.6	8.2	** < 0.001**
	Normal	1.9	2.4	4.1	5.3	3.5	
Current Breastfeeding status	Yes	2.1	3.0	5.0	5.6	4.1	** < 0.001**
	No	5.2	6.4	6.4	12.0	7.9	
Division	Barisal	1.9	5.8	4.9	6.1	4.8	**0.007**
	Chattogram	1.9	3.7	7.6	8.1	5.5	
	Dhaka	3.3	3.4	4.8	6.6	4.9	
	Khulna	1.9	3.0	7.0	4.7	4.3	
	Rajshahi	3.5	2.6	3.9	6.9	4.3	
	Sylhet	1.6	2.7	3.1	5.0	3.2	
Place of Residence	Rural	1.5	2.3	4.4	5.6	3.6	** < 0.001**
	Urban	4.1	5.7	6.8	8.1	6.3	
Wealth Index	Poor	0.7	1.1	3.2	3.9	2.4	** < 0.001**
	Middle	1.5	2.3	5.5	7.5	4.4	
	Rich	4.4	6.1	6.9	8.6	6.7	** < 0.001**
Media Exposure	Yes	3.3	4.4	5.8	7.2	5.4	** < 0.001**
	No	0.9	1.2	4.0	5.1	3.0	
Mother’s Educational Level	No education	0.9	1.5	4.8	6.9	2.8	** < 0.001**
	Primary	1.5	2.8	4.2	4.8	3.4	
	Secondary	3.4	3.7	5.5	7.1	5.2	
	Higher	5.9	7.1	6.3	6.8	6.6	
Father’s Educational Level	No education	1.1	1.4	4.0	5.5	2.9	** < 0.001**
	Primary	2.7	3.7	5.3	6.6	4.9	
	Secondary	3.2	6.3	5.9	9.4	6.4	
	Higher	6.5	6.8	7.3	6.7	6.9	
Mother’s Working Status	Yes	1.8	3.6	4.6	6.1	4.7	0.567
	No	2.6	3.3	5.3	6.7	4.5	
Mother’s Decision-making Capacity	Yes	2.5	3.8	5.8	6.8	5.0	** < 0.001**
	No	2.3	2.3	3.3	4.6	3.0	
Attitude towards Violence	Yes	1.3	1.9	5.5	6.5	3.6	**0.001**
	No	3.0	4.0	5.0	6.4	4.9	
Mother’s Age	15 to 24	1.6	1.7	3.4	4.0	2.8	** < 0.001**
	Greater than 24	3.5	5.5	7.3	9.2	6.7	
Received ANC	Yes	3.1	4.2	5.8	6.7	5.3	** < 0.001**
	No	1.3	1.3	2.8	2.9	1.8	
Initiation of Breastfeeding	Within 1 h	2.7	4.0	6.1	7.3	5.0	**0.007**
	After 1 h	2.1	2.7	4.3	5.9	4.1	
Wanted Child	Yes	2.7	3.4	5.0	6.4	4.6	0.300
	No	1.8	3.3	5.5	6.4	4.2	
Child’s Sex	Female	2.6	3.4	5.5	5.9	4.5	0.870
	Male	2.3	3.3	4.8	6.9	4.6	
Birth Order	First birth	2.7	2.4	3.3	4.7	3.4	** < 0.001**
	Otherwise	2.3	3.9	6.4	7.5	5.2	
Child’s Age	< 18	2.3	2.2	4.5	4.6	3.5	** < 0.001**
	18 +	2.6	4.9	5.9	9.0	5.9	
Vitamin A consumption	Yes	2.8	4.4	5.4	6.9	5.1	** < 0.001**
	No	1.9	2.4	4.8	5.5	3.7	

Table 2 shows that the prevalence of DBM at the household level among the children delivered by CS is more than two times of the children born by normal delivery (8.2% vs. 3.5%) and has a significant impact on DBM (*p*-value < 0.001). While going through the prevalence of DBM by mode of delivery decomposed by survey years, a surge in DBM rate is particularly seen over the year in both CS birth and vaginal birth. The children born by CS delivery face a 10% relative increased risk of DBM from BDHS 2007 to BDHS 2011 (7% vs 7.7%). The relative increase turns to 8% from BDHS 2011 (7.7%) to BDHS 2014 (8.3%) and 3.6% from BDHS 2014 (8.3%) to BDHS 2017 (8.6%) for the children with CS delivery. In the case of children born by normal delivery, a relative increase of 26.3% from BDHS 2007 to BDHS 2011 is evident from the result (1.9% vs 2.4%). However, the prevalence of DBM among children born by normal delivery increased from 2.4% to 4.1% between the time interval of BDHS 2011 and BDHS 2014 with a 70.8% relative increase. Afterwards, the prevalence of DBM faces a 29.3% relative increase from BDHS 2014 to BDHS 2017 (4.1% vs 5.3%). Though the increase of prevalence of DBM in the case of normal delivery is also very acute, the percentage is always lower than the prevalence of DBM for CS delivery in each wave of the survey.

Table 2 also portrays that the children who are breastfed have a lower prevalence of DBM than their counterparts and are significantly associated with DBM. Among the divisions, Chittagong and Dhaka are more vulnerable to the prevalence of DBM than the others (5.5% and 4.9% respectively). Moreover, urban residents and rich subpopulations have higher DBM prevalence than their opposites (6.3% and 6.7% respectively). Surprisingly, households with highly educated mothers and fathers are at increased prevalence of DBM (6.6% and 6.9% respectively). The prevalence of DBM among the mothers in the older age group is more than 2 times than of that of the mothers in the younger age group (2.8% vs 6.7%). Mothers who received ANC are at higher prevalence of facing DBM than their counterparts (5.3% vs. 1.8%). However, the firstborn child is at lower prevalence of being affected by DBM than the others (3.4% vs 5.2%). A child aged more than 18 months has an almost 6% prevalence of DBM whereas the prevalence of DBM in child less than 18 months is only 3.5%. Astonishingly, the prevalence of DBM is lower among the children who are not given vitamin A supplements (3.7%) than their counterparts (5.1%).

### Multivariate analysis

Table 3 is showing the findings obtained from the logistic regression model applied to the pooled data considered in this study. Here, the survey year is considered as a quantitative independent variable for the ease of calculation.

**Table 3:** Adjusted odds ratio (OR) of double burden of malnutrition (DBM) obtained from logistic regression

Covariates and their categories		OR	*p*-value
Year		1.43	** < 0.001**
Mode of Delivery	C-section	2.63	** < 0.001**
	Normal	1	
Year* Mode of Delivery		0.78	**0.002**
Current Breastfeeding status	Yes	0.79	**0.035**
	No	1	
Division	Barisal	1	
	Chattogram	0.97	0.841
	Dhaka	0.79	0.109
	Khulna	0.73	0.061
	Rajshahi	0.87	0.322
	Sylhet	0.62	**0.006**
Place of Residence	Rural	1	0.010
	Urban	1.26	
Wealth Index	Poor	1	
	Middle	1.62	** < 0.001**
	Rich	1.89	** < 0.001**
Media Exposure	Yes	1.03	0.767
	No	1	
Mother’s Educational Level	No education	1	
	Primary	0.97	0.877
	Secondary	1.06	0.738
	Higher	0.83	0.379
Father’s Educational Level	No education	1	
	Primary	1.27	**0.033**
	Secondary	1.35	0.078
	Higher	1.17	0.379
Mother’s Decision-making Capacity	Yes	1.21	0.086
	No	1	
Attitude towards Violence	Yes	0.98	0.874
	No	1	
Mother’s Age	15 to 24	1	** < 0.001**
	Greater than 24	2.07	
Received ANC	Yes	1.63	**0.001**
	No	1	
Initiation of Breastfeeding	Within 1 h	1.15	0.089
	After 1 h	1	
Birth Order	First birth	0.77	**0.017**
	Otherwise	1	
Child’s Age	< 18	1	** < 0.001**
	18 +	1.50	
Vitamin A consumption	Yes	1.03	0.771
	No	1	

**Fig. 3:**
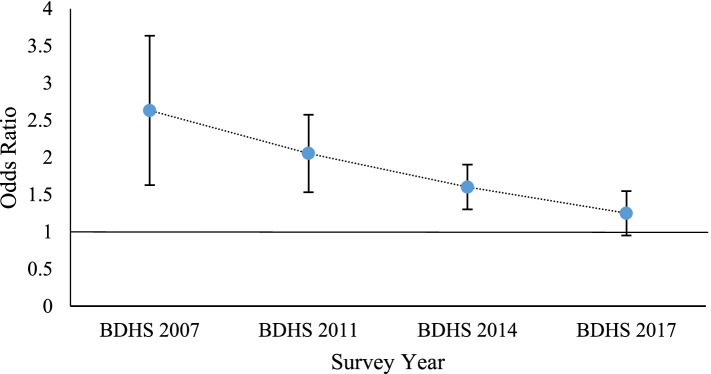
The ORs for DBM for C-section delivery vs. normal delivery by survey year)

**Fig. 4:**
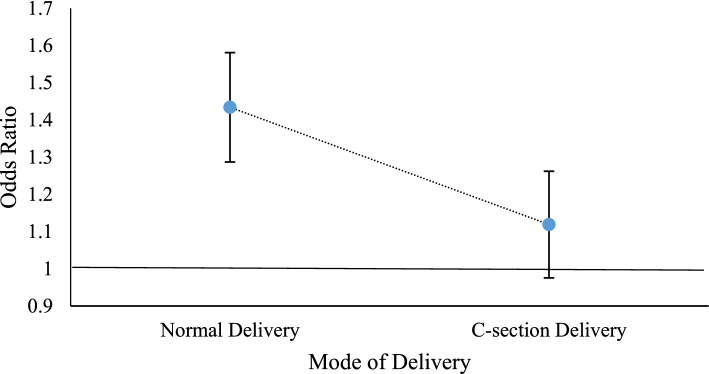
The ORs of DBM for survey year by mode of delivery)

The main effect odds ratio (OR) for survey year and mode of delivery are 1.43 and 2.63 respectively whereas OR for interaction effect is 0.78 and all of them are significant at 5% level of significance. Figure 3 and Fig. 4 are displaying the adjusted OR of DBM for survey years and mode of delivery with corresponding 95% confidence intervals (CIs) where delta method is used in the calculation of OR and corresponding standard error required for CIs [26].

Figure 3 is indicating that the adjusted OR of DBM for CS delivery compared to normal delivery is greater than 1 for each survey year which means that the children born by CS delivery are more likely to be affected by DBM than the children born by normal delivery in every survey years. Moreover, the values of the odds ratio are showing a decreasing pattern with survey year. For instance, the OR of DBM for CS delivery is 2.63 times of the OR of DBM for normal delivery in 2007. Furthermore, the OR of DBM for CS delivery is 1.25 times of the OR of DBM for normal delivery in 2017. That means, the difference in magnitude of the odds ratio is narrowed with the survey year indicating the increased risk of DBM among children born by normal delivery with time.

It is noticeable from Fig. 4 that the adjusted OR for DBM for survey year is greater than 1 for each mode of delivery which suggests that the OR of DBM is increasing with one unit change in time for both normal delivery and CS delivery. However, the OR of DBM is increased by 43% for one unit change in time in case of normal delivery whereas children born by CS delivery have 12% higher odds of DBM with one unit change in time. It stipulates that the risk of DBM is inflaming with time precariously not only for CS delivery but also for normal delivery.

Table 3 is also representing the adjusted effect of other categorical independent variables on DBM which are found significantly associated with the outcome in bivariate analysis. The children who were breastfed at the time of interview are 21% less likely to be affected by DBM at household level than their counterparts. Division has a significant adjusted association with DBM and it is found that the households of Sylhet are 38% less likely to be exposed to DBM compared to Barisal. Wealth index of household has highly significant adjusted effect on DBM and it is noted that the OR shows an increase as the level of wealth index increases where the poor are considered as the reference group (OR = 1.62 and 1.89 for the middle and the rich respectively). Moreover, the households with highly educated fathers have significantly higher odds compared to the households with uneducated fathers (OR = 1.17). Mothers in the older age group have 2.07 times OR of DBM than the mothers aged 15 to 24 years. Babies who obtained early initiation of breastfeeding have a 15% higher odds of DBM than their counter parts. Again, the firstborn children have 23% lower odd of DBM than the other births in the same household and the adjusted effect is significant at 5% level of significance (*p*-value = 0.017). The children aged more than 18 months have 50% higher odd of DBM than their counterparts. Additionally, consumption of vitamin A supplements among children increases the odds of DBM by 3% relative to their opposites. The rest of the variables considered in multivariate analysis such as place of residence, media exposure, educational level of the mother, mother’s decision making capacity, attitude towards violence and ANC reception have an insignificant adjusted impact at 5% level of significance.

## Discussion

The study provides a documentation of the fact that the prevalence of DBM at the household level among mother–child pairs is increasing drastically over time. The study also evinces that a strong association between DBM and mode of delivery is accountable for the boom of this nutrition dilemma and children born by CS delivery are more associated with DBM than the children born by normal delivery. Furthermore, the study reveals that the rate of increase in the prevalence of DBM is steeper for birth by normal delivery rather than CS delivery birth.

The study has revealed an upcoming dread warning in the nutrition system of Bangladesh with an expeditious increase in DBM at the household level over a decade where the prevalence of DBM in several survey years is in line with previous studies [14, 24, 27, 28]. The findings of the study also suggest that delivery by CS can be a potential risk factor behind the increase in the rate of DBM at the household level over the years which is supported by previous literature [24]. It can be possibly explained by gut microbial alteration in small intestines among children due to CS delivery [24]. Less diversity in the intestinal microbiome among infants born by CS delivery results in inflammatory bowel disease and diarrhea that ultimately causes stunting and faltered physical and mental growth [29, 30]. On top of that, overweight mothers are at a higher risk of CS delivery [31] and are associated with late initiation of breastfeeding [32]. Initiation of the cycle of DBM in the household also begins with the inability of mothers going through CS delivery to breastfeedtheir infant according to WHO recommendation because of unconsciousness due to anesthesia during surgery [33].

The study also highlights that the rate of increase of DBM is rapidly rising with time among the households where the childbirth is followed by not only CS delivery but also normal delivery. This kind of expansion is a clear indication of the nutrition transition happening in Bangladesh where dominance of undernutrition coincides with the emergence of obesity [34]. This nutritional transformation describes the pattern of regular diet [4] which is considered as a key driver of DBM [17]. Countries going through such transition shift to western diet which contains fat and high calories ignoring the traditional diet with vegetables and food [35, 36]. This shifting in the dietary pattern is the consequence of increased sale and cost-effectiveness of such ultra-processed foods [17]. Bangladesh already has a traditional diet influenced by rice, sugar and oil rather than fruits or vegetables. Besides, the intake of fast food and soft drinks in the country is remarkably going up [37, 38]. Evidence shows that, the regular consumption of junk foods and soft drinks and physical inactivity among the youth of Bangladesh leads to a higher risk of overweight and obesity [38]. Maternal overweight or obesity increases the risk of not only the pre-term birth but also the low birth weight [39]. Additionally, consumption of ultra-processed food in the first thousand days of life causes a vulnerable contribution to stunting among children [17]. Concisely, change in dietary pattern and physical inactivity are now instigating the lifestyle pattern and hence the enigmatic situation of DBM turns up for both mode of delivery.

The study also depicts that several demographic, socio-economic and health-related variables are associated with DBM. The findings report the existence of regional variation in the prevalence of DBM. Two main metropolitan areas, Chittagong and Dhaka, are most affected by DBMwhich is in line with previous literature [40]. The rapid growth of urbanization in these two areas of the country may affect the lifestyle of the residents which may refrain them from physical activity and active travel that leading them to obesity. Additionally, urban design with insufficient hygiene may cause water-borne diseases and several infections among children and hence under-nourishment among them [4]. Thus imbalanced and unplanned urbanization in divisions of Bangladesh fuels the paradoxical problem addressed in the study. The effect of DBM among rich households is very common according to the findings of the study which is supported by previous studies [27, 41]. This pattern can be interpreted by the high intake of ultra-processed and energy-dense food among the rich in South Asian countries [27] which not only increases the risk of being overweight among the adults but also raises the under-nutrition rate among the children [27]. A strong association between DBM at the household level and paternal education is reported in this study which matches with previous evidence [40]. In a patriarchal society set-up in Bangladesh, improvement in the educational status of fathers imposes a powerful impact on adopting a healthy lifestyle in household which impedes the way of booming DBM. The study sheds light on the fact that older maternal age has a significant impact on the rise of DBMconforming to other studies in past [14, 24, 41]. A sedentary lifestyle and reduced metabolic rate in older age of mothers increase the risk of obesity among them [41]. In addition, the unpopularity of postpartum resolution of weight gain in Bangladesh [24] gears up the overweight issue in the older age of mothers which instigates the rate of DBM. The result carries a manifestation that breastfeeding practice can be a protective factor against increased DBM mirroring the findings of previous literature [27]. The production of milk inside the mother’s body during breastfeeding is a calorie-burning process in the postpartum period which helps the mother to lose pregnancy weight more quickly [42, 43]. Additionally, breast milk is the ideal food for infants being rich in fat, protein and vitamins with antibodies being a protector for the children from the risk of asthma, allergy, and several inflectional and non-communicable diseases and thus lowering the malnutrition among children in the early stage of life [44]. As breastfeeding not only helps mothers in losing weight but also enriches children with proper micronutrients, it has a positive association with lowering DBM prevalence. Surprisingly, the study demonstrates that the mothers who received at least one antenatal visit are more vulnerable to DBM which opens a new sphere of association between DBM and maternal healthcare-seeking behavior. The finding of the study that the household with first birth face less prevalence of DBM than their counterparts is consistent with the previous study conducted by Hauqe et al. [27]. It can be possibly explained by the immense sensitivity of new mothers towards their first births’ health and diet and their less obese form due to younger age during first birth. The result also demonstrates that household having children in the higher age group also has a higher prevalence of DBM that is embraced by earlier findings in Bangladesh [27, 45]. The plausible explanation of such findings can be linked to the notion that breastfeeding practice reduces in older age group and therefore children start to face a lack of ideal diet and mothers gain weight due to a reduction in losing calories [27, 45]. Consequently, the oddity of overweight/obese mother and undernourished child pairs at the household level arises among the higher age groupof children.

The study bears a powerful message that DBM at household level is highly associated with mode of delivery over time which has not been revealed in any other study up till now to the best of our knowledge. It extracted data from a nationally representative survey which provides reliable estimates on aspects related to maternal and child health. However, the study does not provide any causal relationship between the outcome variable and the covariates due to the use of different waves of cross-sectional data. The study may have some recall bias which has been mitigated to some extent by considering only the mother–child pairs where the child is born in the last three years preceding the survey. More exploration on several critical factors related to food insecurity, dietary patterns of households and child care practice are rigorously needed. The confounding effect of several attributes on delivery mode such as income of respondents, access to health-care facility can add a new dimension to the study which is absent in the current one due to unavailability of information in BDHS survey. The study can be extended further by investigating the triple burden of malnutrition under the same roof in LMICs and finding room for improvement in the nutritional paradigm.

DBM can be viewed from a different perspective which provides the policymakers the scope of double returns by addressing interventions for both undernutrition and overnutrition. WHO provides a plan of double duty action [4] that aims to eradicate the paradox of the new nutritional era by controlling overweight related issues among adults and undernutrition related problems among children concurrently. However, the policymakers must focus on frameworks that are designed to counsel the mothers who went through CS delivery on postpartum health care and the diet of children. A healthy food system and sustainable diet plans based on dietary guidelines for all ages need to be promoted for all sub-groups of the population by coherent programs. Multisectoral interactional programs on support for breastfeeding practice, healthcare coverage, and implementation of nutrition education are highly required. Conclusively, the increase of DBM with time passively opens a new horizon of improvement for policymakers by synergistically fighting against all forms of malnutrition at all ages with integrated policies and initiatives.

## Data Availability

The Bangladesh Demographic and Health Survey (BDHS) data used in this study are available in the DHS website and it can be downloaded without any cost from https://dhsprogram.com/data/available-datasets.cfm.

## Abbreviations

BDHS: Bangladesh Demographic and Health Survey
CS: Caesarean Section
DBM: Double Burden of Malnutrition
LMIC: Lower-and-middle-income-countries
NCD: Non-communicable Disease
SDG: Sustainable Development goal
WHO: World Health Organization
